# Excessive Sensitivity to Uncertain Visual Input in L-DOPA-Induced Dyskinesias in Parkinson’s Disease: Further Implications for Cerebellar Involvement

**DOI:** 10.3389/fneur.2014.00008

**Published:** 2014-02-04

**Authors:** James K. R. Stevenson, Chonho Lee, Bu-Sung Lee, Pouria TalebiFard, Edna Ty, Kristina Aseeva, Meeko M. K. Oishi, Martin J. McKeown

**Affiliations:** ^1^Kinsmen Laboratory of Neurological Research, Department of Neuroscience, University of British Columbia, Vancouver, BC, Canada; ^2^School of Computer Engineering, Nanyang Technological University, Singapore, Singapore; ^3^Department of Electrical and Computer Engineering, University of British Columbia, Vancouver, BC, Canada; ^4^Pacific Parkinson’s Research Centre, University Hospital, University of British Columbia, Vancouver, BC, Canada; ^5^Department of Electrical and Computer Engineering, University of New Mexico, Albuquerque, NM, USA

**Keywords:** l-DOPA-induced dyskinesias, Parkinson’s disease, dynamical system models, visually guided tracking, visual uncertainty

## Abstract

When faced with visual uncertainty during motor performance, humans rely more on predictive forward models and proprioception and attribute lesser importance to the ambiguous visual feedback. Though disrupted predictive control is typical of patients with cerebellar disease, sensorimotor deficits associated with the involuntary and often unconscious nature of l-DOPA-induced dyskinesias in Parkinson’s disease (PD) suggests dyskinetic subjects may also demonstrate impaired predictive motor control.

**Methods:** We investigated the motor performance of 9 dyskinetic and 10 non-dyskinetic PD subjects on and off l-DOPA, and of 10 age-matched control subjects, during a large-amplitude, overlearned, visually guided tracking task. Ambiguous visual feedback was introduced by adding “jitter” to a moving target that followed a Lissajous pattern. Root mean square (RMS) tracking error was calculated, and ANOVA, robust multivariate linear regression, and linear dynamical system analyses were used to determine the contribution of speed and ambiguity to tracking performance.

**Results:** Increasing target ambiguity and speed contributed significantly more to the RMS error of dyskinetic subjects off medication. l-DOPA improved the RMS tracking performance of both PD groups. At higher speeds, controls and PDs without dyskinesia were able to effectively de-weight ambiguous visual information.

**Conclusion:** PDs’ visually guided motor performance degrades with visual jitter and speed of movement to a greater degree compared to age-matched controls. However, there are fundamental differences in PDs with and without dyskinesia: subjects without dyskinesia are generally slow, and less responsive to dynamic changes in motor task requirements, but in PDs with dyskinesia, there was a trade-off between overall performance and inappropriate reliance on ambiguous visual feedback. This is likely associated with functional changes in posterior parietal–ponto–cerebellar pathways.

## Introduction

Prediction is a fundamental component of motor control. For instance, when catching a baseball it is necessary to predict where the ball will be at a given instant and how much force its impact will generate in order to prepare the hand for the catch. Central to motor prediction is the forward model, which enables prediction of the sensory effects of movement (1). Substantial evidence indicates that humans use forward models to predict the sensory consequences of their own actions (1–6), as well as to predict the dynamics of objects in the external environment (4, 7–11). Furthermore, forward models of object dynamics are necessary to guide visuo-motor coordination tasks, and can even override observed kinematic feedback (12, 13).

Predictive forward modeling becomes even more imperative as the reliability of visual feedback is compromised, for example in dim lighting, or disease states such as Parkinson’s disease (PD) where the visual system may be affected (14). Normally, human subjects account for the degree of sensory uncertainty during motor performance by de-weighting their reliance on sensory feedback when it is ambiguous (1, 6, 15–20), and instead more heavily rely on predictive forward models (6, 18, 20). However, when subjects are unable to use predictive motor control, the motor response no longer anticipates sensory feedback but rather reacts to it in an uncoordinated manner (21–24).

Motor performance in PD, at least in the early stages of the disease, is greatly improved by pharmacotherapy, and l-DOPA remains the gold standard of treatment in PD (25). However, l-DOPA-induced dyskinesias (LIDs) – excessive choreoathetoid involuntary movements – are a relatively common side effect of l-DOPA of which peak-dose LIDs are the most common type (26). Though LID pathophysiology remains unclear, behavioral studies suggest that rather than being a purely motor phenomenon, LIDs may be associated with deficits in sensorimotor control (27–29). For example, dyskinetic subjects have demonstrated increased variation in tracking velocity during a visually guided tracking task that was significantly reduced when visual feedback was withdrawn, suggesting an exaggerated motor response to sensory input (27). As dyskinetic subjects are often unaware of their involuntary movements (30), and have been shown to underestimate the distance their limb has moved (28), a component of sensorimotor deficits associated with LIDs may be attributed to impaired predictive motor control. For instance, a mismatch between predicted movement and actual movement may drive dyskinetic subjects to compensate for the sensory discrepancy with excessive movement. Deficits in predictive motor control are typically seen in diseases of the cerebellum (31–36), yet such deficits have also been demonstrated in PD (37, 38). There is evidence to support cerebellar involvement in LIDs (39), and altered activity and plasticity in the prefrontal cortex in dyskinetic subjects (40, 41) may contribute to altered sensorimotor control in LIDs.

If inadequate predictive motor control is an underlying feature of dyskinetic subjects’ motor performance, then a heightened reliance on sensory feedback should be especially prominent in conditions where healthy subjects rely more heavily on predictive forward models, such as when confronted with ambiguous visual feedback (6). Accordingly, we hypothesized that dyskinetic subjects would demonstrate an impaired ability to de-weight ambiguous visual feedback during a visually guided tracking task. We have purposely chosen a motor adaptation task, whereby subjects had to adapt to changing sensory information. We have recently demonstrated that overall, PD subjects are susceptible to sensory uncertainty during visually-guided tracking (42), but in that study we did not dichotomize dyskinetic and non-dyskinetic subjects. We have since recruited additional PD subjects while employing the same tracking task to assess the reliance on uncertain visual feedback of dyskinetic and non-dyskinetic PD (NDPD) subjects. As previous work has demonstrated linear dynamical system (LDS) models to be a sensitive marker of motor performance in PD (42, 43), here we use LDS models in addition to quantifying tracking error to assess tracking performance. By extracting the decay rate parameter from the LDS models during ambiguous tracking, we quantified subjects’ relative reliance on uncertain visual feedback.

## Materials and Methods

### Subjects

The Ethics Board of the University of British Columbia approved the study and all subjects gave written, informed consent. We recruited 19 patients with probable PD according to diagnostic criteria (44) and 10 age-matched control subjects without active neurological disorders. Exclusion criteria included known PD with dementia. PD subjects were Hoehn and Yahr stage 1–3 (45), and 9 subjects were dyskinetic PD (DPD) subjects and 10 were NDPD subjects. We did not screen subjects for the presence of depression or anxiety, however we excluded PD subjects with dementia and all subjects were cognitively able to follow the instructions and complete the tasks. Subject characteristics are shown in Table 1. All patients had overnight withdrawal of medications before the study for at least 12 h for l-DOPA and 18 h for dopamine agonists. We calculated the converted l-DOPA daily dosage as 100 mg l-DOPA = 125 mg of controlled-release l-DOPA, which was then added to the equivalents of dopamine agonists to give the l-DOPA equivalent daily dosage (LEDD), where 100 mg of l-DOPA = 1 mg of pramipexole, 6 mg of ropinirole, 10 mg of bromocriptine, 75 mg of l-DOPA plus entacapone. The presence of peak-dose LIDs was assessed up to 1.5 h after the l-DOPA challenge, where subjects received the equivalent of their morning l-DOPA dose given in the immediate release form. Peak-dose LIDs were defined by the presence of involuntary choreiform movements in any of the head/neck, trunk, and upper limbs of variable duration and in some cases were accompanied by dystonia. LID severity was assessed according to the Goetz Dyskinesia Rating Scale (46), and all DPD subjects had mild LID that were of minimal severity and did not interfere with voluntary motor acts (Goetz score = 1). Disease severity was assessed according to the Unified Parkinson’s Disease Rating Scale (UPDRS) motor score in the off medication state.

**Table 1:** **Subjects’ characteristics**.

Subject	Age	Disease duration	Motor exam UPDRS	Converted daily l-DOPA dosage (mg)	Other Parkinson’s medications	Type of LID chorea (C) dystonia (D)	l-DOPA equivalent daily dose (mg)
**DPD**							
D1	65	22	65	650	Rop, amant	C, D	750
D2	64	7	42	880	Entac, amant	C	1173.3
D3	68	13	51	660	Entac	C	880
D4	65	15	57	720	Entac	C, D	960
D5	66	5	45	1020	None	C	1020
D6	64	4	22	1280	Pram	C	1580
D7	51	7	37	800	Bromo	C, D	1000
D8	55	13	40	640	Pram, amant	C, D	665
D9	75	8	47	600	None	C	600
DPD (mean ± SD)	63.7 ± 7	10.44 ± 5.8	45.11 ± 12.3	805.56 ± 223.33			958.7 ± 296.27
**NDPD**							
ND1	63	5	8	320	Pram	None	620
ND2	68	4	19	400	None	None	400
ND3	64	9	69	860	None	None	860
ND4	59	9	14	740	None	None	740
ND5	45	4	11	780	None	None	780
ND6	65	9	51	640	Entac, pram	None	1003.3
ND7	63	10	54	800	Pram	None	1000
ND8	66	7	22	640	Rop	None	673.3
ND9	62	5	31	400	None	None	400
ND10	59	12	47	400	Pram	None	775
NDPD (mean ± SD)	61.4 ± 6.4	7.4 ± 2.8	32.6 ± 21.2	598 ± 200.3			725.2 ± 211.3
Control (mean ± SD)	61.6 ± 7.9						
*p* Value	0.75	0.16	0.14	0.047			0.062

*Rop, ropinirole; pram, pramipexole; amant, amantadine; bromo, bromocriptine; entac, entacapone*.

### Study paradigm

The large-amplitude tracking task used here has been previously described (42). Briefly, a Lissajous figure was presented on a screen measuring 1.62 m × 1.22 m with a red circular target (12 cm in diameter) in the center of the screen. Subjects stood approximately 55 cm in front of the screen, and tracked the moving target with their index finger, requiring movement about the wrist, elbow, and shoulder joints. We tested subjects in the standing position in order to facilitate larger amplitude movements more representative of everyday life that are often precluded in imaging studies. Additionally, evidence suggests LIDs may be of greater amplitude in the standing compared to the sitting position (47). In the baseline trials the target smoothly followed the Lissajous path, either at a slow tracking speed (average speed of 56.2 cm/s) or a fast tracking speed (average speed of 78.3 cm/s). In subsequent visually ambiguous conditions, the target jittered about the path while maintaining the path’s overall trajectory. In the ambiguous tracking conditions, subjects were instructed to attempt not to chase the jitter, but rather to attempt to track the desired target’s position, which maintained the overall Lissajous trajectory. Four levels of visual ambiguity were tested (0, 0.03, 0.05, 0.07) – representing the jitter root mean square (RMS) amplitude with respect to screen height (0, 0.0191, 0.0318, and 0.0445°), at two speeds, giving a total of eight conditions. The jitter was obtained by first starting with random Gaussian noise sampled at the frame rate of 60 Hz. Because we did not want excessive discontinuities in the visual pattern caused by high-frequencies, we then low-passed the random series at 20 Hz. Each condition was tested in three different trials, where a trial consisted of 30 s of tracking, a 12 s rest, followed by 30 s of tracking. The order of the trials began with the slow non-ambiguous condition followed by the fast non-ambiguous condition. The order of the remaining six ambiguous conditions was randomly selected. This same trial order of all eight tracking conditions was then repeated for the second and third trials. The trial order was the same for every subject. Subject DPD 9 was an exception and completed two trials of each condition due to complaints of fatigue. PD subjects performed this motor task in the morning when in the “off” medication state, and after a break for lunch subsequently repeated the task in the “on” medication state that same day.

### Quantification of manual tracking

We used a Polhemus Fastrak (Polhemus, Colchester, VT, USA) six-degrees-of-freedom electromagnetic tracking system to record subject tracking. A stylus sensor was held in the palm of the subjects’ dominant hand and secured with tape. The tip of the stylus was aligned with the tip of the index finger in order to record subjects’ index finger position. A time series for displacement was recorded in the *x*, *y*, and *z* directions, and data was recorded at 10 Hz. We performed a robust linear regression analysis on the *x* and *y* sensor data during non-ambiguous tracking to determine the optimum affine transformation to map the sensor data coordinates to the Lissajous figure coordinates. We subsequently applied the same transformation to the ambiguous conditions on a subject-by-subject basis.

### Quantification of tracking performance

Root mean square tracking error was calculated by subtracting the processed *x* and *y* sensor data of the index finger from the *x* and *y* target position along the baseline track, squaring the result for each time point, taking the mean for the squared values for each trial, and computing the square root of the result.

Analysis of motor performance using LDS models is being increasingly utilized in sensorimotor studies (16, 48–51) and has been previously used to rigorously characterize tracking performance in PD (42, 43). We computed LDS models of subjects’ tracking using system identification techniques (52) and extracted the decay rate parameter, which describes how quickly tracking performance returns to equilibrium after a perturbation. Intuitively, a higher decay rate can be considered akin to the tighter suspension of a sports car: tighter turning on a good road may be desirable, but when an uneven gravel (noisy) road is encountered, the ability to smooth out the bumps (i.e., de-weight the noise) is diminished. Thus during ambiguous tracking, higher decay rates can intuitively be interpreted as a greater response to ambiguous visual feedback (see Figure S1 in Supplementary Material). The natural logarithm of the decay rates were used to make the results more Gaussian distributed and this was subsequently used in all statistical analyses.

### Statistical analyses

**Figure 1:**
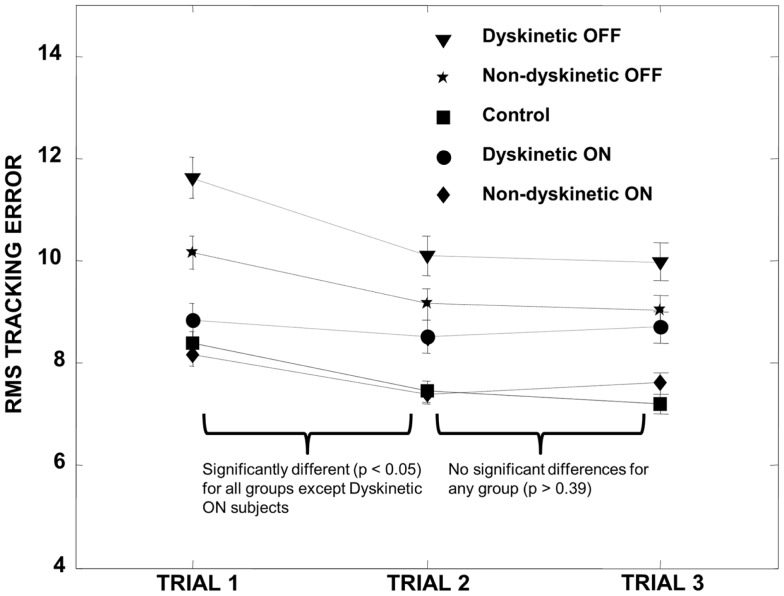
**Training effect**. All groups demonstrated a significant decrease in RMS error between trial 1 and trial 2 except for the DPD ON group (where the decrease was not significant), and the RMS error subsequently stabilized for all groups between trials 2 and 3. We therefore omitted trial 1 data in all subsequent calculations. Error bars represent the standard error.

MatLab (The MathWorks Inc., MA, USA) was used for all statistical analyses. In order to control for a training effect between tracking trials, we first performed paired *t*-tests on the pooled RMS error of all groups (DPD and NDPD off and on medication and control) between trial sets 1 and 2 and trial sets 2 and 3. We observed a training effect between trial set 1 and 2 that stabilized between trial set 2 and 3 (Figure 1), and trial 1 data were therefore omitted from all subsequent data analysis to ensure we were not examining motor learning in our visually guided tracking task but rather the effect of visual uncertainty after learning had occurred and stabilized.

We used mixed-model analysis of variance (ANOVA) to assess different effects on both overall RMS error as well as decay rate. In the first instance, we examined the effect of ambiguity, tracking speed, and drug status (i.e., ON or OFF l-DOPA), PD group (i.e., DPD or NDPD), and subject number as factors in the ANOVA. Since the same PD subjects were assessed before and after l-DOPA, ambiguity, tracking speed, and drug status were considered within subject factors and subject number was nested within the PD group factor.

To compare the PD subjects with Normal subjects, we also used a mixed-model analysis of variance (ANOVA), where ambiguity, tracking speed, group (i.e., N, DPD ON or OFF l-DOPA, or NDPD ON or OFF l-DOPA) and subject number were used as factors in the ANOVA. As before, ambiguity and tracking speed were considered within subject factors and subject number was nested within the group factor. We then repeated the above two ANOVA analyses with log(decay rate) instead of RMS error.

In order to examine the relationship between UPDRS and the effect of ambiguity on tracking performance, we calculated Spearman’s rank correlation coefficients between UPDRS and the difference in RMS error between the non-ambiguous and maximum ambiguous tracking conditions for each PD group, as well as between UPDRS and decay rate in each of the ambiguous tracking conditions. In order to better visualize the results of the ANOVA, a robust multivariate regression analysis was also performed, using RMS error or log(decay rate) as the dependent variable, and speed and ambiguity as the independent variables. Regression coefficients were obtained to indicate the portion of dependent variable explained by speed and ambiguity amplitude. Quality of LDS models’ was assessed by the Akaike information criterion (AIC) with a model quality score based on a trade-off between matching the data well and penalizing the use of an excessive number of model parameters. Significance for all comparisons was declared at *p* < 0.05. We estimated the stability of the regression coefficients and the group-wise RMS and log(decay rate) values by leave-one-out validation.

**Figure 2:**
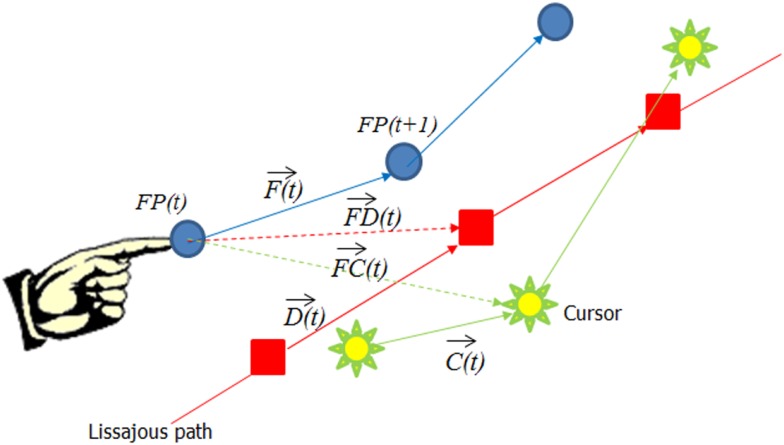
**The decomposition of the finger movement vector into different constituent vectors**. The blue arrow represents the finger velocity $\left[\vec{F}\left(t\right)\right]$, the green arrow the cursor velocity $\left[\vec{C}\left(t\right)\right]$, the red arrow the desired velocity $\left[\vec{D}\left(t\right)\right]$, the green dotted arrow from the finger position to the cursor $\left[\vec{F}C(t)\right]$, and the red dotted arrow represents the vector from the finger to the desired point $\left[\vec{F}D(t)\right]$.

In order to further evaluate what features in visual input influenced finger movement, we decomposed finger velocity into its projection along different vectors. As shown in Figure 2, we looked at Finger velocity $\vec{F}\left(t\right)$ [i.e., the vector from FP(*t*) to FP(*t* + 1)], depicted as a Blue arrow; the cursor movement on the screen $\vec{C}\left(t\right)$: green arrow, the desired velocity along the Lissajous path, $\vec{D}(t)$; the path from finger point to cursor point, $\vec{\text{FC}}(t)$ green dotted arrow; and $\vec{\text{FD}}(t)$ the path from the finger to the desired point: red dotted arrow. We used the “robustfit” function of MatLab to estimate the coefficients of a multivariate linear regression equation:

$$\left(\;\begin{array}{c} F_{x}\left(1\right)\\ F_{y}\left(1\right)\\ F_{x}\left(2\right)\\ F_{y}\left(2\right)\\ \ldots \end{array}\;\right)=F_{o}+\left(\;\begin{array}{cccc} FC_{x}\left(1\right) & FD_{x}\left(1\right) & C_{x}\left(1\right) & D_{x}\left(1\right)\\ FC_{y}\left(1\right) & FD_{y}\left(1\right) & C_{y}\left(1\right) & D_{y}\left(1\right)\\ FC_{x}\left(2\right) & FD_{x}\left(2\right) & C_{x}\left(2\right) & D_{x}\left(2\right)\\ FC_{y}\left(2\right) & FD_{y}\left(2\right) & C_{y}\left(2\right) & D_{y}\left(2\right)\\ \ldots & \ldots & \ldots & \ldots \end{array}\;\right)\;\left(\;\begin{array}{c} \beta_{1}\\ \beta_{2}\\ \beta_{3}\\ \beta 4 \end{array}\;\right)+\in$$

We then compared DPD subjects before medication and after medication (i.e., D-pre vs. D-post), NDPD before and after medication (ND-pre vs. ND-post), as well as the difference in finger movement trajectories with and without noise for all groups of patients.

## Results

Subjects’ characteristics are shown in Table 1. There were no significant differences between age, UPDRS, disease duration, and LEDD (*p* > 0.05), though converted daily l-DOPA dosage was significantly higher for the DPD group. Analysis of RMS error between trials revealed that there was a significant decrease in the pooled RMS error between trial set 1 and trial set 2 (*p* < 0.00001) that stabilized between trial sets 2 and 3 (*p* = 0.7). The individual groups’ RMS error by trial is shown in Figure 1.

The results of the mixed-model ANOVA tests on RMS error are shown in Tables 2 and 3. When comparing DPD and NDPD subjects, ambiguity and tracking speed were significant independent factors on RMS error, as well as the interaction terms between ambiguity and tracking speed, PD group and drug status. When normal subjects were included in the analysis (Table 3), ambiguity, tracking speed, and group were all significant factors, as well as the interaction terms between ambiguity, speed, group, and subject. Similarly, when determining the effects on log(decay rate), ambiguity and speed were significant when comparing DPD and NDPD (Table 4), as well as the interaction between l-DOPA and PD group (Table 4). When control subjects were included in the analysis, significances were seen in the main effects of ambiguity, speed, and group, as well as the interaction effects of ambiguity and speed and group (Table 5).

**Table 2:** **Mixed-model analysis of variance (ANOVA) table to assess different effects on overall RMS error in PD subjects**.

RMS: dyskinetic vs. non-dyskinetic PD					
Source	Sum sq.	df	Mean sq.	*F*	Prob > *F*
Ambiguity	1764.544	1	1764.544	246.2379	1.5 × 10^−11^
Speed	125.1352	1	125.1352	76.27172	2.9 × 10^−11^
l-DOPA	10.50281	1	10.50281	2.544744	0.12
PD group	2.378259	1	2.378259	0.841198	0.37
Subject (PD group)	48.0629	17	2.827229	0.588631	0.87
Ambiguity × speed	18.34189	1	18.34189	19.12748	1.8 × 10^−05^
Ambiguity × l-DOPA	28.59159	1	28.59159	29.81618	1.2 × 10^−07^
Ambiguity × PD group	39.13004	1	39.13004	5.460503	0.03
Ambiguity × subject (PD group)	121.8222	17	7.166014	7.472938	1.6 × 10^−14^
Speed × l-DOPA	3.5044	1	3.5044	3.654495	0.057
Speed × PD group	0.791581	1	0.791581	0.257766	0.62
Speed × subject (PD group)	52.20574	17	3.070926	3.202455	3.7 × 10^−05^
l-DOPA × PD group	0.15373	1	0.15373	0.014268	0.90
l-DOPA × subject (PD group)	183.1669	17	10.77452	11.236	6.7 × 10^−22^
Error	215.7589	225	0.958929		
Total	4069.522	303			

*Root mean square error was taken as the dependent variable and the effect of ambiguity, tracking speed, and drug status (i.e., ON or OFF l-DOPA), PD group (i.e., DPD or NDPD), and subject number were used as factors in the ANOVA. Subject number was nested within the PD group factor, since the same PD subjects were assessed before and after l-DOPA. RMS, root mean square; Sum sq., sum of squares; df, degrees of freedom; Mean sq., mean squares; *F*, *F* statistic; Prob, probability*.

**Table 3:** **Mixed-model analysis of variance (ANOVA) table to assess different effects on overall RMS error in all subjects**.

RMS: all subjects					
Source	Sum sq.	df	Mean sq.	*F*	Prob > *F*
Ambiguity	1975.723	1	1975.723	451.6872	1.9 × 10^−24^
Speed	152.2342	1	152.2342	149.8938	1.0 × 10^−23^
Group	21.47974	4	5.369934	3.660012	0.01
Subject (group)	63.08918	43	1.46719	1.445266	0.058
Ambiguity × speed	18.13801	1	18.13801	25.40773	9.1 × 10^−7^
Ambiguity × group	99.69423	4	24.92356	5.69799	9.0 × 10^−4^
Ambiguity × subject (group)	188.0861	43	4.374096	6.127235	1.1 × 10^−20^
Speed × group	10.30256	4	2.57564	1.562198	0.20
Speed × subject (group)	70.89532	43	1.648728	2.309539	3.7 × 10^−5^
Error	170.6168	239	0.713878		
Total	4707.803	383			

*Root mean square error was the dependent variable and ambiguity, tracking speed, group (i.e., N, DPD ON or OFF l-DOPA, or NDPD ON or OFF l-DOPA), and subject number were used as factors in the ANOVA, with subject number nested within the group factor. RMS, root mean square; Sum sq., sum of squares; df, degrees of freedom; Mean sq., mean squares; *F*, *F* statistic; Prob, probability*.

**Table 4:** **Mixed-model analysis of variance (ANOVA) table to assess different effects on overall log(decay rate) in PD subjects**.

Log(decay rate): dyskinetic vs. non-dyskinetic PD					
Source	Sum sq.	df	Mean sq.	*F*	Prob > *F*
Ambiguity	8.197592	1	8.197592	109.5405	0
Speed	0.483603	1	0.483603	6.462162	0.01
l-DOPA	0.14046	1	0.14046	1.876896	0.17
PD group	0.062757	1	0.062757	0.365946	0.56
Subject (PD group)	18.32016	17	1.077656	14.40021	0
Ambiguity × speed	0.084005	1	0.084005	1.122517	0.29
Ambiguity × l-DOPA	0.107382	1	0.107382	1.434896	0.23
Ambiguity × PD group	0.422255	1	0.422255	5.642388	0.01
Speed × l-DOPA	0.052067	1	0.052067	0.695753	0.40
l-DOPA × PD group	1.581883	1	1.581883	21.13796	7.5 × 10^−6^
Error	15.04207	201	0.074836		
Total	61.64793	227			

*The factors used were identical to that of Table 2, only log(decay rate) was used as opposed to overall RMS error as the dependent variable. Sum sq., sum of squares; df, degrees of freedom; Mean sq., mean squares; *F*, *F* statistic; Prob, probability*.

**Table 5:** **Mixed-model analysis of variance (ANOVA) table to assess different effects on overall log(decay rate) in all subjects**.

Log(decay rate): all subjects					
Source	Sum sq.	df	Mean sq.	*F*	Prob > *F*
Ambiguity	168.2415	1	168.2415	652.8348	0
Speed	0.644047	1	0.644047	4.313921	0.03
Group	2.767405	4	0.691851	3.262799	0.02
Subject (group)	9.117819	43	0.212042	1.420009	0.05
Ambiguity × speed	1.165972	1	1.165972	6.882016	9.2 × 10^−3^
Ambiguity × group	5.921167	4	1.480292	5.74404	8.5 × 10^−4^
Ambiguity × subject (group)	11.08149	43	0.257709	1.521099	0.027
Speed × group	0.573569	4	0.143392	1.339341	0.270
Speed × subject (group)	4.603654	43	0.107062	0.631919	0.964
Error	40.4921	239	0.169423		
Total	275.5562	383			

*The factors used were identical to that of Table 3, only log(decay rate) was used as opposed to overall RMS error as the dependent variable. Sum sq., sum of squares; df, degrees of freedom; Mean sq., mean squares; *F*, *F* statistic; Prob, probability*.

The differences in RMS error between non-ambiguous and maximum ambiguous tracking conditions were not significantly correlated with UPDRS scores at either tracking speed for either dyskinetic subjects or for non-dyskinetic subjects and *p* > 0.05.

**Figure 3:**
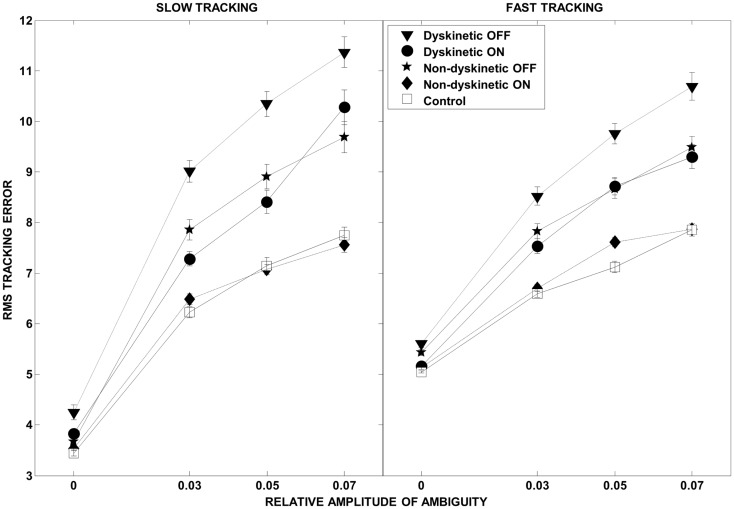
**Root mean square error as a function of visual ambiguity and tracking speed**. Differences in RMS error in the low tracking speed condition (left panel) and high tracking speed condition (right panel) are shown. Error bars were estimated by leave-one-out-validation.

The overall effect of increasing ambiguity and speed on overall tracking performance, and the l-DOPA effect, is illustrated in Figure 3. As expected, there were increases in RMS error with both speed and visual ambiguity. DPD subjects had the highest error, which was partially ameliorated by l-DOPA. In NDPD subjects, after medication, the tracking error approached that of control subjects.

**Figure 4:**
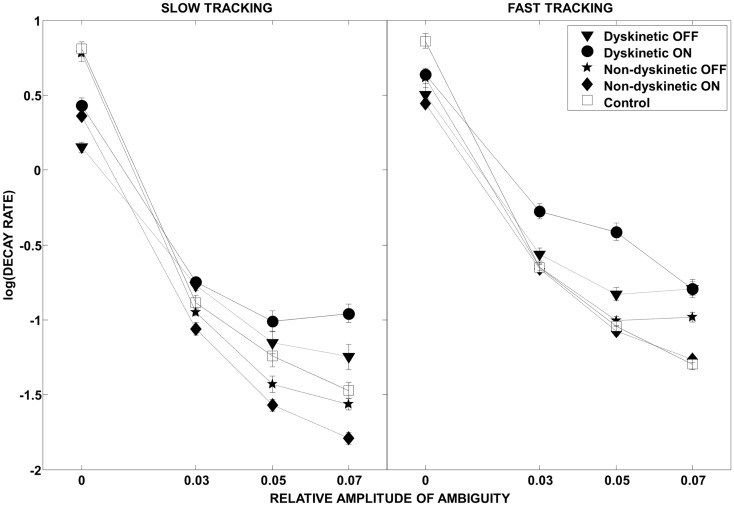
**Log(decay rate) as a function of visual ambiguity and tracking speed**. Differences in RMS error in the low tracking speed condition (left panel) and high tracking speed condition (right panel) are shown. Error bars were estimated by leave-one-out-validation.

The effect of visual ambiguity on the log of decay rate is shown in Figure 4. At slow speeds and higher levels of ambiguity, NDPD subjects had lower log(decay rates) than controls (left panel). However, at higher tracking speeds the NDPD subjects had similar or higher log(decay rates) as controls. In contrast, DPD subjects had higher values for log(decay rate) at high ambiguity levels at both speeds, a situation not ameliorated by medication.

**Figure 5:**
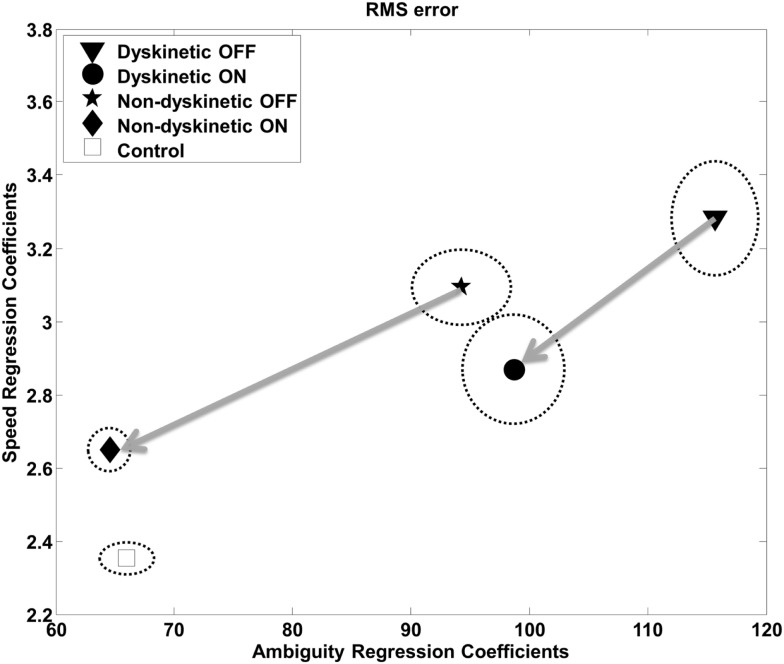
**Regression analysis – RMS**. The relative contribution of increasing target ambiguity and speed to RMS error is demonstrated. The design matrix for the regression included tracking speed, ambiguity columns, as well as nuisance covariates corresponding to individual subject and overall mean. The units can be considered arbitrary. The vertical and horizontal radii of the ellipses represent the standard error of the regression coefficients as estimated by leave-one-out validation. The arrows are from the same PD subjects OFF medication to ON medication, and thus show the effects of l-DOPA medication.

The regression analysis illustrates the relative contribution of increasing ambiguity and speed to RMS error (Figure 5) and log(decay rate) (Figure S1 in Supplementary Material) by group. The speed and ambiguity regression coefficients captured by the model were highly significant for all groups (*p* < 10–5), and the between group differences in both speed and ambiguity regression coefficients were also highly significant (*p* < 10–5). Figure 5 demonstrates that increasing tracking speed and ambiguity contributed to the RMS error of DPD OFF subjects significantly more than for all other groups. Additionally, the susceptibility to speed and visual ambiguity is not normalized with medication for DPD ON subjects, but is roughly normalized for NDPD ON subjects. Figure S1 in Supplementary Material suggests that log(decay rate) is significantly affected by visual ambiguity in PD, but especially so for DPD subjects. l-DOPA had less of an effect on the sensitivity of log(decay rate) to tracking speed in DPD compared to NDPD subjects.

The Akaike’s final prediction error (FPE) and AIC used to assess the LDS models from ambiguous tracking conditions revealed robust tracking models. The means and standard deviations of the estimated LDS models’ FPE and AIC scores were ≤3.1 ± 2.0 and ≤1.8 ± 0.4 respectively, for all groups across all conditions, which is indicative of high model quality/fit. Furthermore, there were few outliers in the FPE and AIC values indicating validity of the modeling framework across subject groups.

**Figure 6:**
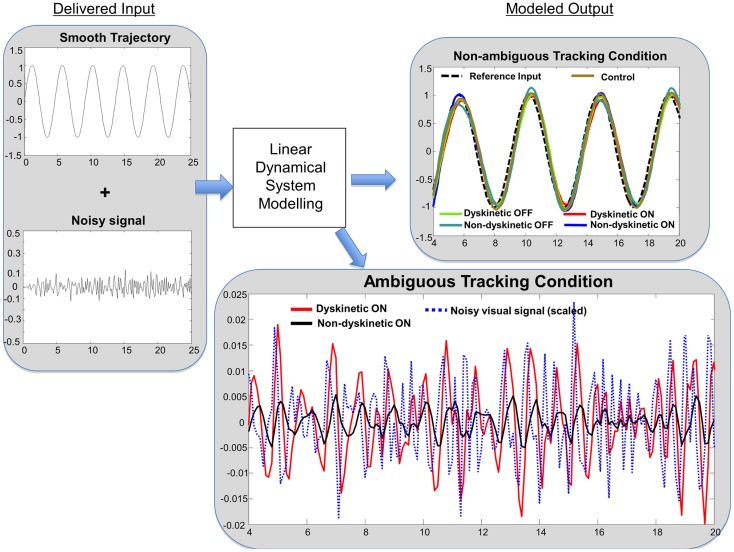
**Linear dynamical system model simulation results**. Left panel: tracking input is a combination of a smooth sinusoidal reference trajectory and bandpass filtered white noise. Right panel: subjects modeled tracking output in a smooth non-ambiguous tracking condition (*upper box*) and in a “noisy” ambiguous tracking condition (*lower box*). In the upper box, the modeled tracking output of a representative subject from each group is shown. The subjects’ output in non-ambiguous conditions is similar across groups. In the lower box, the effect of “noisy” ambiguous input on modeled output of a dyskinetic and a non-dyskinetic subject is shown. The increased decay rate of dyskinetic subjects on medication leads them to attempt to track the noise more significantly. The noisy visual signal is reduced to 15% of the actual magnitude for visualization purposes.

In order to get an intuitive interpretation of the significantly different decay rates, we interrogated typical models from each group (i.e., models with eigenvalues close to the mean for each group) with one-dimensional sinusoidal inputs and additive noise similar to the experiment to determine the predicted tracking performance. Ideal tracking performance would occur in systems that ignore the noisy input and faithfully maintain sinusoidal tracking. Consistent with the RMS error results, the sinusoidal tracking improved with post-medication models in both dyskinetic and non-dyskinetic subjects. However, consistent with the statistical results, the dyskinetic model had an impaired ability to ignore the noisy visual cue, and was excessively reliant on noisy ambiguous visual feedback (Figure 6).

We observed the significant differences in the decomposition of the finger tracking data, depending upon whether or not jitter was present (Figure S2 in Supplementary Material). For only the DPD (D-pre and D-post) group, we found that contribution of $\vec{\text{FD}}(t)$ – the vector from finger position to the desired position – increased (*p* = 0.036 and *p* = 0.026), while the contribution of $\vec{D}\left(t\right)$ – the desired trajectory – decreased when jitter was present (*p* = 0.002 and *p* = 0.005).

## Discussion

We examined the ability of 9 dyskinetic and 10 NDPD subjects, as well as that of 10 age-matched control subjects, to de-weight uncertain sensory feedback and instead rely more heavily on predictive motor control during visually guided tracking. The relative contributions of increasing target ambiguity and speed (examined independently) to the RMS tracking error were the greatest for DPD subjects off medication (Figure 3). As expected, l-DOPA medication improved overall tracking performance for both PD groups, as evidenced by reduced RMS error with medication (Figure 3).

Dyskinetic subjects’ gross motor performance was worse in the Parkinsonian state than in the “on” medication state despite the presence of LIDs. This is consistent with the presence of mild LIDs experienced by DPD subjects that did not interfere with the completion of motor tasks, and the increased movement in the on-state compared to the Parkinsonian state that enabled improved overall motor performance. However in dyskinetic subjects, improved overall tracking performance came at a price: they were also more responsive to and reliant on non-informative visual cueing (Figure 6, right lower panel). We interpret our results in the context of established performance trade-offs in control theory, in which controllers that produce exceptionally fast, high-performance tracking under ideal circumstances are also extremely poor at disturbance rejection (that is, they experience high sensitivity to external or un-modeled noise processes). In a biological context, this is indicative of a system that relies more heavily on ambiguous sensory feedback and less on predictive motor control. This interpretation is further supported by the decomposition of the finger velocity $\vec{F}\left(t\right)$. For DPD group only (i.e., D-pre and D-post), the contribution of the desired trajectory $\vec{D}\left(t\right)$, to finger velocity, $\vec{F}\left(t\right)$, was significantly reduced during jitter cases, implying that finger movement velocity was significantly degraded by jitter in these subjects. Interestingly, FD(*t*), i.e., where their finger was to where it should have been, significantly increased in the DPD group only, possibly reflecting an compensatory corrective motor movement after the realization that they had been misled by the jitter. The trends we observed in the LDS are consistent with the trends we observed through the regression analysis. The LDS model separates the contribution of the output due to the desired trajectory from the contribution of the output due to the additive jitter. The decreased reliance on $\vec{D}\left(t\right)$ in DPD subjects is consistent with the increased decay rate in the LDS – that is, the subjects are following the noise more than they were in the no-noise case.

It is important to note that the LDS models utilized here are deterministic. The numerical algorithm used to identify the LDS model, in fact, minimizes the residual between the actual output and the predicted output, in essence capturing as much information as possible from the input–output relationship and leaving only white noise. What would we see if the only differences between DPD and NDPD were that the DPD subjects had the same tracking performance but superimposed dyskinesias that were truly random fluctuating movements? The parameters of the models would be the same, but the residuals of the model, which reflect the part of the movement not accurately captured by the deterministic model, would be much higher in the DPD case. Yet we observed the opposite: the model residuals were not significantly higher in the DPD subjects (as reflected by the lack of significant differences in their model scores) and the parameters of the model were appreciably different. In fact, this raises an important issue, that a key interpretation of our findings is that LIDs may not be “random” at all as is normally assumed, but a deterministic response to various endogenous and exogenous stimuli that is normally appropriately de-weighted. This may explain why increasing sensitivity to stimuli such as that seen in anxiety (53), or increased vigilance due to cognitive or motor load (54) may increase dyskinesias.

Normally, forward models are used to predict sensory feedback, and the predicted feedback is subsequently compared to actual feedback when it becomes available after an inherent delay (55, 56). The difference between the actual and predicted sensory feedback is known as the sensory discrepancy or error, which is then used to update the forward model and in turn improve motor performance (1, 56). In fact, the concept of forward modeling has been extended from predicting the sensory consequences of movement to predicting the external environment (4, 7–13). For example, evidence indicates that human subjects utilize forward models of visual cues (11), of target motion during interception tasks (12, 13), and of the physical laws of gravitational acceleration (4, 9, 15). We quantified RMS tracking error as the difference between the subjects’ index finger position and the target position along the smooth Lissajous path at any given time point. During the non-ambiguous conditions of our tracking task, the sensory discrepancy is likely minimal as the predicted sensory feedback relating the subjects’ index finger position and the target position would be congruent, which is supported by the lack of differences in RMS tracking error between the groups in the baseline conditions (Figure 3). However, in the ambiguous tracking conditions the sensory discrepancy would be large due to the ambiguous jitter of the target. Human subjects have been shown to reliably predict the mean perturbation delivered from a variable distribution in reaching tasks (50, 57), and to do so according to Bayesian inference (17).

Though we did not explicitly test the use of Bayesian statistics in this study, the strategy of more heavily weighting the mean jitter amplitude and de-weighting the instantaneous uncertain jittering position of the target in order to predict the desired tracking position, corresponds to the optimal motor response in our task that minimizes RMS tracking error.

As expected, in our study, normal controls had the lowest overall tracking error of all groups (Figure 3). However, Figure 4 provides interesting insight into how this is achieved. During slow tracking and in high ambiguous situations, NDPD subjects had even lower decay rates than controls, suggesting that they were robust to the ambiguity – so much so that they excessively de-weighted the (still partially meaningful) visual information. However, in the high speed tracking condition, it becomes more critical to de-weight the visual information and the originally sluggish approach of the NDPD becomes the appropriate response – this is why NDPD patients ON medication and controls had essentially the same decay rates. These observations are consistent with other studies demonstrating that NDPD subjects do not overly respond to visual feedback (58), and that healthy human subjects internally account for sensory uncertainty and de-weight uncertain feedback during motor performance (1, 18, 20). In contrast, in both slow and fast tracking conditions, the DPD subjects demonstrated excessively high decay rates (Figure 4), implying faster dynamics, even though this resulted in excessive overall tracking error (Figure 2).

Thus, the inability of DPD subjects to de-weight ambiguous visual data that we observed may be based on excessive sensitivity to discrepancies between a (accurate) forward model and sensory feedback and/or an impaired forward model. The effect of l-DOPA medication may provide insight on this. In addition to reducing overall tracking error (Figure 3), l-DOPA medication made overall tracking error less susceptible to tracking speed and visual ambiguity (Figure 5), but had minimal effect on the log(decay rate)’s sensitivity to tracking speed and visual ambiguity (Figure S1 in Supplementary Material). If we assume that decay rate is related to corrective sub-movements and hence responses to discrepancy between models, this would imply that l-DOPA largely improves forward model accuracy (and hence reduced RMS error’s sensitivity to visual ambiguity and speed) while having minimal effect on the dynamics of the response to the errors between the forward model and sensory estimates (Figure 5).

A possible functional neuroanatomical correlate of the inability of DPD subjects to de-weight ambiguous visual feedback demonstrated in the present study is inadequate predictive cerebellar forward modeling. There is growing evidence of functional cerebellar changes occurring in PD (59–64) and in DPD (39) that supports this possibility. Furthermore, the cerebellum is known to have an integral role in predictive motor control, and predictive deficits that lead subjects to excessively respond to feedback are typically seen in cerebellar disease (21, 24, 31–34). Extensive evidence supports the use of forward models in human subjects (2–6), and neuroimaging, electrophysiology, and transcranial magnetic stimulation (TMS) studies provide strong evidence for the role of the cerebellum in forward modeling (31, 65–75). Interestingly, evidence from neuroimaging studies demonstrates significantly increased cerebellar activity in conditions of mismatch between predicted and actual feedback (66), and the degree of mismatch imposed by temporal delays has been correlated with cerebellar activity (76). Further evidence indicates that the cerebellar climbing fiber-Purkinje cell synapse may signal the error between the predicted and actual sensory feedback (71, 72, 77–80).

In addition to the cerebellum, the posterior parietal cortex (PPC) is believed to have an important role in predictive motor control (65). The role of the PPC in making on-line corrections (a process that requires forward models) during movement has been demonstrated in patients with lesions to this area and through the use of TMS (81, 82). TMS applied to the PPC of healthy human subjects prevented them from making fast on-line corrective movements to a target perturbation in a reaching task when vision of their arm was occluded, and they instead continued to reach to the initial target (81). As DPD subjects in our study were found to be overly responsive to the ambiguous visual feedback (as opposed to unresponsive PPC subjects), this may argue against altered PPC function explaining our results. Moreover, PPC stimulation has been related to motor awareness (83), and interestingly DPD patients can be unaware of their involuntary movements (30). Nonetheless it is possible that altered PPC activity contributed to the impaired predictive motor control of dyskinetic subjects, and as the PPC and cerebellum have reciprocal neuroanatomical connections (84, 85), it is likely that these two structures work together in using forward models to guide motor performance. Given that frontal “executive” dysfunction is well described in PD (86), it is tempting to speculate whether or not impaired frontal lobe dysfunction may contribute to the our observation of impaired forward models in PD. While this may explain, at least in part, the differences between PD subjects as a whole and controls, we do not believe that it could explain the differences between NDPD and DPD subjects we observed.

There is increasing evidence that although dyskinesias are present when DPD subjects are on medications, changes in motor function persist off medication (42). For example, Figure 3 demonstrates that DPD subjects OFF medication were significantly worse in overall tracking compared to NDPD subjects OFF medication. Animal models of PD suggest that unnatural pulsatile stimulation of dopaminergic receptors, occurring with intermittent dosing of l-DOPA, may induce plastic changes that contribute to the development of LIDs (87, 88). Interestingly, younger patients are more prone to developing LIDs (89), and this may be related to a greater degree of plasticity occurring in the younger brain. Additionally, neurochemical changes related to LIDs (90) are not limited to the basal ganglia. Nimura and colleagues (91) demonstrated that the binding potential of the cerebellar sigma receptors was positively correlated with LID scores but not with disease severity of PD patients undergoing pallidotomies; while Koch and colleagues (39) have demonstrated altered cerebellar plasticity in DPD subjects using TMS. Furthermore, we have found behavioral differences that differentiate dyskinetic from non-dyskinetic subjects in the off medication state that may be related to altered cerebellar functioning (42). Thus, the dyskinetic brain may exhibit altered cerebellar plasticity that manifests functionally as inadequate predictive motor control. Direct neuroanatomical pathways connecting the basal ganglia and the cerebellum have been found in primates (92, 93), providing a direct route for the administration of l-DOPA to interact with altered cerebellar structures.

There are a number of potential limitations to our study. First, there was a trend toward greater disease severity of dyskinetic than NDPD subjects, though the difference was non-significant (Table 1). Nonetheless, in order to fully address this, we examined the relationship between UPDRS and the increase in RMS between the non-ambiguous and maximum ambiguous tracking conditions, and no significant correlation for either DPD or NPDP subjects was found. Thus, the worsening of motor performance with increasing visual ambiguity was not associated with disease severity. Furthermore, UPDRS was not significantly correlated with decay rate for either PD groups, except for in the slow tracking condition (ambiguity level = 0.03), which was the only ambiguous tracking condition that lacked significant differences in mean decay rate between groups. Second, in addition to testing while on medication, we also tested PD subjects in the practically defined off medication state with 12 h of l-DOPA withdrawal and 18 h for dopamine agonists, and subjects were symptomatic upon study commencement. We note that this method of examining the practically defined off medication state in PD is universally utilized (94, 95), though we acknowledge that this may not reflect a truly depleted dopaminergic state. Furthermore, non-motor complications can occur with the off medication state (96), as well as pain that can be experienced by DPD patients (97), and such non-motor complications were not accounted for in this study. However, none of the subjects complained of pain and none of the subjects experienced non-motor complications requiring them to stop the study. Third, dyskinetic subjects can experience postural instability while on medication and experiencing LIDs (98), and we did not quantify postural instability between groups. However none of the subjects from either PD group complained of postural problems, and the lack of difference in overall accuracy in the baseline tracking condition of our task indicates that PD subjects were able to perform the task while standing equally well as healthy control subjects, and suggests any potential differences in postural instability did not significantly affect motor performance. Fourth, we did not examine potential differences in visual acuity between dyskinetic, non-dyskinetic, and control subjects. However, once again a lack of difference in RMS error in the baseline non-ambiguous conditions of the task suggests that any differences in visual acuity were not great enough to impact baseline motor performance. Fifth, it is theoretically possible that l-DOPA affected DPD and NDPD subjects with respect to eye movements. However, we note that (99) we found no changes in smooth pursuit gain during dose-related on–off fluctuations, so believe that this was not a factor here.

In conclusion, we demonstrate that DPD subjects are significantly more susceptible to visually ambiguous sensory input during a visually guided tracking task, and that the improvement in overall tracking performance with l-DOPA medication comes at a price for DPD subjects: an increased reliance on ambiguous visual feedback. The results indicate inadequate weighting of predictive motor control in DPD, which may be a significant contributor to pathophysiology of LIDs. We discuss possible cerebellar dysfunction in DPD as a neuroanatomical substrate of inadequate weighting of predictive motor control.

## Conflict of Interest Statement

The authors declare that the research was conducted in the absence of any commercial or financial relationships that could be construed as a potential conflict of interest.

## Supplementary Material

The Supplementary Material for this article can be found online at http://www.frontiersin.org/Journal/10.3389/fneur.2014.00008/abstract

Figure S1 **Regression analysis – log(decay rate)**. The regression was the same as for Figure 4, only the dependent variable used in the regression was log(decay rate).

Figure S2 **Regression weights of the vector decomposition in Figure 2, comparing the situations with and without “jitter**.”
