# Supplementary figures and images for: Excessive Sensitivity to Uncertain Visual Input in L-DOPA-Induced Dyskinesias in Parkinson’s Disease: Further Implications for Cerebellar Involvement
Source: Front Neurol. 2014 Feb 4;5:8. doi: 10.3389/fneur.2014.00008 (PMC3912458; doi:10.3389/fneur.2014.00008)

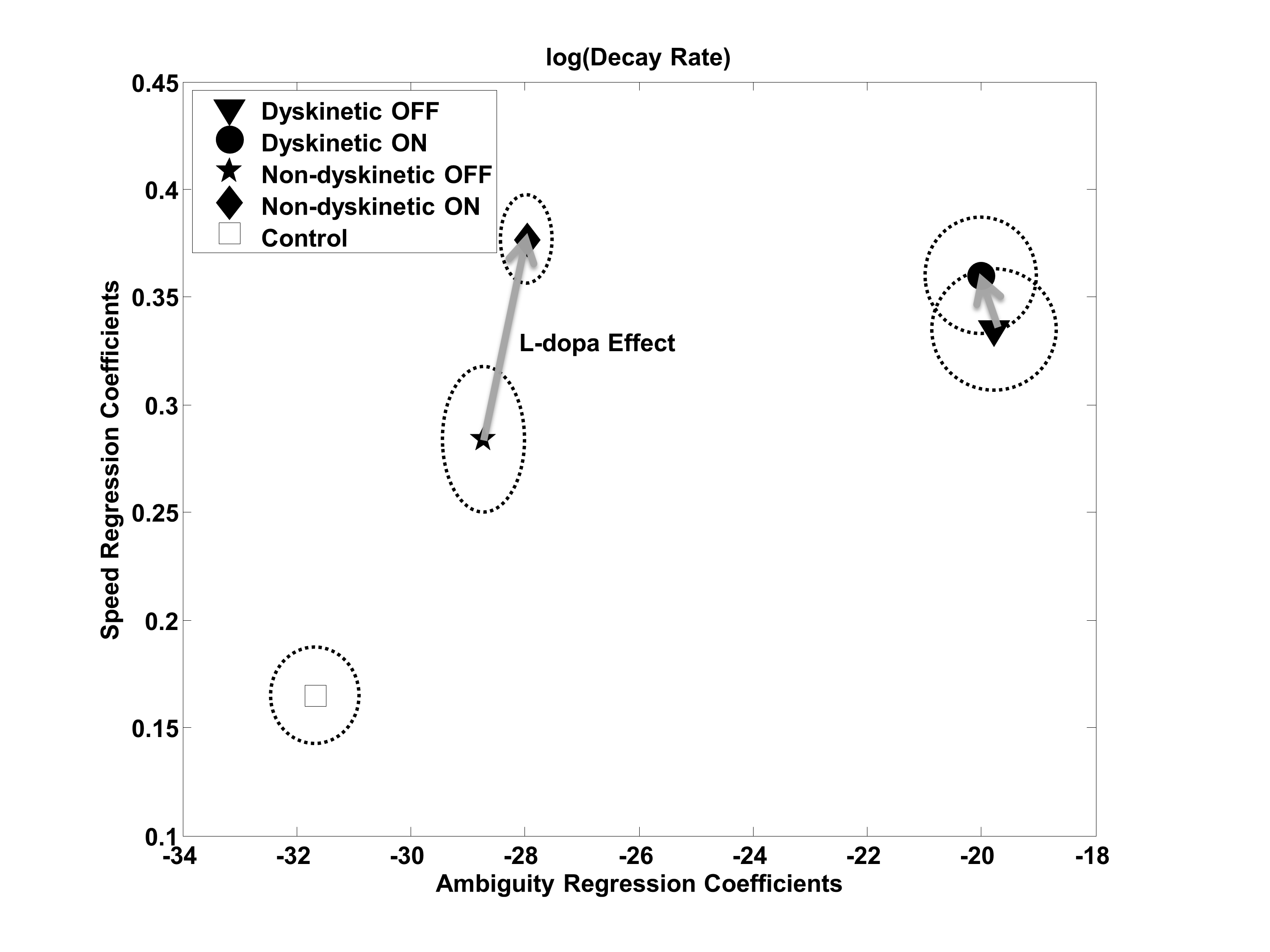

Supplement: Figure S1 — Regression analysis – log(decay rate). The regression was the same as for Figure 4, only the dependent variable used in the regression was log(decay rate). [file 77206_McKeown_Presentation1.ZIP › Supplementary Figure 1.tif]

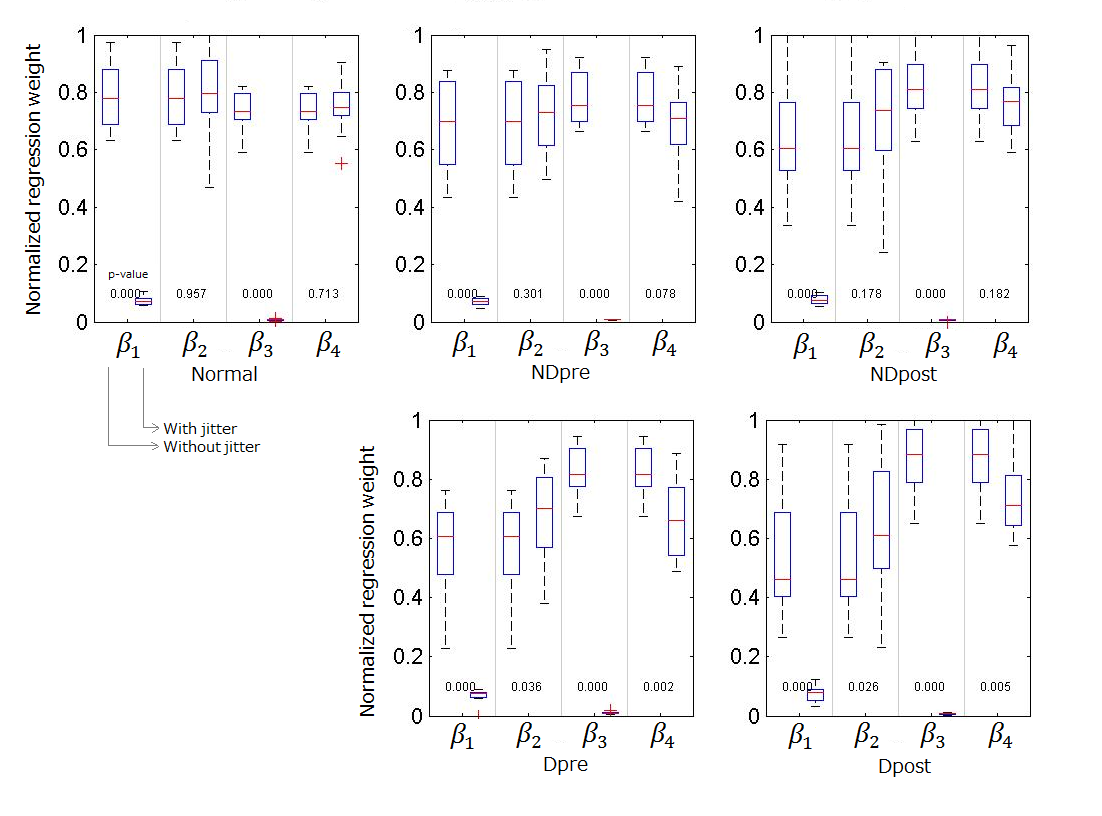

Supplement: Figure S1 — Regression analysis – log(decay rate). The regression was the same as for Figure 4, only the dependent variable used in the regression was log(decay rate). [file 77206_McKeown_Presentation1.ZIP › Supplementary Figure 2.tif]
